# Characterization of Changes in Serum Anti-Glycan Antibodies in Crohn's Disease – a Longitudinal Analysis

**DOI:** 10.1371/journal.pone.0018172

**Published:** 2011-05-06

**Authors:** Florian Rieder, Rocio Lopez, Andre Franke, Alexandra Wolf, Stephan Schleder, Andrea Dirmeier, Anja Schirbel, Philip Rosenstiel, Nir Dotan, Stefan Schreiber, Gerhard Rogler, Frank Klebl

**Affiliations:** 1 Department of Internal Medicine I, University of Regensburg, Regensburg, Germany; 2 Department of Pathobiology, Lerner Research Institute, Cleveland Clinic Foundation, Cleveland, Ohio, United Sates of America; 3 Department of Gastroenterology, Cleveland Clinic Foundation, Cleveland, Ohio, United Sates of America; 4 Department of Quantitative Health Sciences, Cleveland Clinic Foundation, Cleveland, Ohio, United Sates of America; 5 Institute of Clinical Molecular Biology and Department of General Internal Medicine, Christian-Albrechts-University, Kiel, Germany; 6 Glycominds Ltd., Lod, Israel; 7 Departement of Internal Medicine, Clinic for Gastroenterology and Hepatology, University Hospital Zuerich, Zuerich, Switzerland; University of Nebraska Medical Center, United States of America

## Abstract

**Introduction:**

Anti-glycan antibodies are a promising tool for differential diagnosis and disease stratification of patients with Crohn's disease (CD). We longitudinally assessed level and status changes of anti-glycan antibodies over time in *individual* CD patients as well as determinants of this phenomenon.

**Methods:**

859 serum samples derived from a cohort of 253 inflammatory bowel disease (IBD) patients (207 CD, 46 ulcerative colitis (UC)) were tested for the presence of anti-laminarin (Anti-L), anti-chitin (Anti-C), anti-chitobioside (ACCA), anti-laminaribioside (ALCA), anti-mannobioside (AMCA) and anti-*Saccharomyces cerevisiae* (gASCA) antibodies by ELISA. All patients had at least two and up to eleven serum samples taken during the disease course.

**Results:**

Median follow-up time for CD was 17.4 months (Interquartile range (IQR) 8.0, 31.6 months) and for UC 10.9 months (IQR 4.9, 21.0 months). In a subgroup of CD subjects marked changes in the overall immune response (quartile sum score) and levels of individual markers were observed over time. The marker status (positive versus negative) remained widely stable. Neither clinical phenotype nor NOD2 genotype was associated with the observed fluctuations. In a longitudinal analysis neither changes in disease activity nor CD behavior led to alterations in the levels of the glycan markers. The ability of the panel to discriminate CD from UC or its association with CD phenotypes remained stable during follow-up. In the serum of UC patients neither significant level nor status changes were observed.

**Conclusions:**

While the levels of anti-glycan antibodies fluctuate in a subgroup of CD patients the antibody status is widely stable over time.

## Introduction

The diagnosis of inflammatory bowel disease (IBD) and the differentiation between ulcerative colitis (UC) and Crohn's disease (CD) is currently based on the combination of clinical, laboratory, radiological, endoscopic and histopathologic criteria [1]. However, in about 15% of colitis patients a definitive diagnosis cannot be made, a disease category termed indeterminate colitis (IC). In addition, CD is characterized by the frequent occurrence of complicated disease behavior, defined as fistulae or stenoses, and the need for CD-related surgery in a high proportion of patients [2], [3], [4].

Serological markers linked to CD, such as anti-*Saccharomyces cerevisiae* (ASCA), anti-*Pseudomonas*-associated sequence I2 (anti-I2), outer membrane porin C (OmpC) of *Escherichia coli* antibodies and antibodies against the bacterial flagellin cBir1 (Anti-cBir1) have been extensively investigated for diagnosis and disease stratification [5], [6]. The most recently described serum markers directed against microbial antigens are anti-glycan-antibodies. A panel of antibodies consisting of anti-*Saccharomyces cerevisiae* antibodies (gASCA), anti-mannobioside carbohydrate antibodies (AMCA), anti-laminaribioside carbohydrate antibodies (ALCA), anti-chitobioside carbohydrate antibodies (ACCA), anti-laminarin carbohydrate antibody (Anti-L) and anti-chitin carbohydrate antibody (Anti-C) has been reported in several independent cohorts to show a high discriminatory capacity for CD versus UC and association with and prediction of complicated CD behavior [7], [8], [9], [10], [11], [12], [13].

Despite a large number of cross sectional studies and a growing number of prospective studies in patient cohorts examining the utility of anti-glycan antibodies and other serum markers for diagnosis, disease stratification and prediction [5], [6], [7], [8], [9], [10], [11], [12], [13], [14], [15] strikingly limited information is available on stability of antibody levels or antibody status (positive versus negative) over time. Most existing investigations determine stability of markers by facilitating one single sample per patient but this single-point cross-sectional study design makes claims about stability and potential influencing factors on level changes conflicting [7], [9], [16], [17], [18]. Whereas some data are available about level and status changes of serum markers over time in individual patients [11], [14], [19], [20], [21], [22], [23], [24], all studies suffer from low patient numbers and/or short follow-up times. No publication exists assessing the stability of the novel anti-glycan antibodies in individual patients over time.

In contrast to this lack of information, knowledge in this field is crucial not only for interpreting existing studies but also for the design of future prospective trials that can ultimately lead to routine use of these biomarkers in clinical practice. The aim of this study was to fill these information gaps by I) defining the extent of level changes of anti-glycan antibodies in individual patients over time, II) interrogating potential associations of clinical factors and genotypes with marker fluctuations, III) performing a longitudinal analysis following marker levels correlated with distinct clinical events over time and IV) investigating, if the accuracy of the marker panel to differentiate UC from CD and association with complicated CD behavior changes over time.

## Methods

### Patient population

We performed a longitudinal cohort study among adult IBD patients. All IBD in- and outpatients seen at our tertiary referral center between 2000 and 2006 were considered for participation in the study. The diagnosis of CD and UC was made based on clinical, radiographic, endoscopic and histopathologic criteria according to Stange et al. [1], [25].

Inclusion criterion for this study was the presence of more than one serum sample per individual IBD patient during the disease course. The samples were collected at arbitrary visits to our hospital. Clinical data including age at diagnosis, body mass index (BMI), gender, date of sample procurement, date and type of first complication and surgery, medication and disease location were obtained or updated, respectively, for each single visit and time point of sample procurement separately by the treating physician of the IBD unit. Collected data were transferred and stored in a secure coded anonymized database for analysis. In July 2007, all patient charts and the database were reviewed and updated for the data points mentioned above without knowledge of the antibody values. The treating IBD physician determined CD activity based on the criteria included in the Crohn's disease activity index (CDAI) at the time of the patient visit and patients were grouped in active (CDAI>150) and non-active disease (CDAI<150), without assigning a specific CDAI point value. This patient cohort represents a subgroup of the previously reported cross-sectional study [11].

Signed informed consent was obtained from all participants. The ethics committee of the University of Regensburg approved the study.

### Serological analysis

After procurement of the blood samples the serum was separated by centrifugation and kept frozen at −80°C until use. All sera were analyzed at the same time for the levels of gASCA IgG, ALCA IgG, ACCA IgA, AMCA IgG, Anti-L IgA and Anti-C IgA in a blinded manner as previously described [11]. All assays were performed by ELISA following the manufacturer's conditions (Glycominds, Ltd; Lod, Israel). The optical density is directly proportional to the amount of bound antibody. Results were expressed as ELISA units (EU), which is a value relative to a calibration serum sample. The cut-off values were used as previously determined in a larger cross sectional analysis using ROC curves for 90% specificity of CD versus a large control group consisting of UC, other inflammatory GI-diseases, non-GI diseases and healthy controls [11]: ACCA 90 EU, Anti-L 120 EU, Anti-C 50 EU, ALCA 60 EU, AMCA 100 EU, gASCA 50 EU.

For the level of immune response of the serum-antibodies and for analysis of association with certain phenotypes quartile scores for each serologic marker were calculated, as previously described [7], [9], [11], [19]. Briefly, for each antibody patients whose antibody levels were in the first, second, third and fourth quartile of the distribution were assigned a quartile score of 1, 2, 3 or 4, respectively. By adding individual quartile scores for each glycan antigen a semi-quantitative quartile sum score (QSS, range 6–24) representing the cumulative quantitative immune response towards all six antigens for each patient was obtained. In other words the QSS semiquantitatively depicts the overall serologic immune response taking into consideration the levels of all tested antibodies per sample. The QSS at the time of first sample procurement was used as a reference for the longitudinal follow-up analysis.

CRP was recorded as positive (values>0.5 mg/l) or negative (values<0.5 mg/l).

### Determination of NOD2 genotypes of the IBD patients

Standardized TaqMan® SNP Genotyping Assays (Applied Biosystems, Foster City, USA) were used to determine the genotype of the three main CD-associated NOD2 variants SNP 8, 12 and 13 (rs2066844, rs2066845 and rs5743293). All processed data were written to and administered by a previously described database-driven laboratory information management system (LIMS) [26]. Duplicate or related samples were identified and excluded from the analyses, using algorithms implemented in the LIMS. All SNPs had a high call rate (> = 90% in cases and controls), were not monomorphic (minor allele frequency <1% in cases or controls), and did not deviate from Hardy-Weinberg equilibrium (HWE) in the control sample (PHWE>0.001).

### Phenotypical characteristics of IBD patients

The IBD physician assessed the IBD patients for disease phenotype at arbitrary visits during the disease course. Patient demographics at time of first sample procurement can be seen in **Table 1**. For the purpose of this study complicated disease behavior in CD patients was defined as the occurrence of fistulae or stenoses before or during follow-up. We additionally distinguished internal penetrating from perianal fistulizing disease. Furthermore, we examined the need for CD-related surgery during the follow-up period. CD-related surgery included 23 patients that had surgeries other than bowel resection, 64 patients that had bowel resection only and 67 that had both types of surgeries during follow-up.

**Table 1:** Cohort Characteristics.

Factor	CD	UC
	(N = 207)	(N = 46)
Female, n (%)	103 (49.8)	18 (39.1)
Mean age at 1st sample (years) (SD)	35.3 (12.1)	38.0 (12.5)
Mean BMI (kg/m^2^) (SD)	23.6 (5.1)	24.3 (4.7)
Mean age at diagnosis (years) (SD)	28.6 (11.9)	33.1 (12.6)*
Median disease duration at time of first sample (months) (P25, P75)	52.7 (9.2, 122.9)	40.9 (12.9, 97.6)
Median follow-up after first sample (months) (P25, P75)	17.4 (8.0, 31.6)	10.9 (4.9, 21.0)*
Location		
Ileum Involvement	178 (86.0)	----
Proctitis/Left Colon	----	11 (24.4)
Subtotal/Pancolitis	----	34 (75.6)
CRP>0.5 mg/l any time during FU‡	142 (69.3)	25 (54.4)
Behaviour (Montreal classification) Ŧ		
B1	40 (19.4)	---
B1p	20 (9.7)	---
B2	50 (24.3)	---
B2p	17 (8.3)	---
B3	48 (23.3)	---
B3p	31 (15.1)	---
IBD related surgery (%)	154 (74.4)	---
NOD2 Genotype †		
R702W C>T		
CC	1 (0.5)	0 (0.0)
CT	32 (17.0)	1 (2.3)
TT	155 (82.5)	42 (97.7)
G908R G>C		
CC	1 (0.5)	0 (0.0)
GC	12 (6.1)	2 (4.6)
GG	184 (93.4)	42 (95.5)
L1007fsinsC -/C		
C/C	7 (3.6)	0 (0.0)
-/C	43 (21.9)	3 (6.8)
-/-	146 (74.5)	41 (93.2)
All SNPs		
wt	107 (56.9)	37 (86.1)*
mut	81 (43.1)	6 (13.9)
Number of serology tests per subject		
2	76 (36.7)	29 (63.0)
3	56 (27.1)	6 (13.0)
4	26 (12.6)	5 (10.9)
≥5	49 (23.7)	6 (13.0)

BMI, body mass index; IBD, inflammatory bowel disease; CD: Crohn's disease; UC: Ulcerative colitis.

P25, P75: 25th and 75th percentiles; SD: standard deviation.

*p<0.05 versus other patient group.

‡ 2 CD patients did not have any CRP measures during follow-up.

Ŧ One patient did not have clinical information regarding the occurence of fistula.

† Complete NOD2 Genotype information was available for 188 CD and 43 UC patients.

### Statistical analysis

Descriptive statistics were computed for all variables. These include means, standard deviations and percentiles for continuous factors and frequencies for categorical variables. Lin's concordance correlation coefficients were estimated to assess correlation between first and maximal change samples. Associations of level and status changes with clinical phenotypes were evaluated using logistic regression models and adjustments for total follow-up time were done. To study changes in antibody levels, linear mixed models were built; time since diagnosis, use of immunosuppressants, active disease, occurrence of complication, and IBD-related surgery were included in the models. An unstructured covariance matrix was used to model within-subject correlation due to repeated measures. The sensitivity, specificity, positive and negative predictive values were estimated to assess whether the validity of each marker in diagnosing CD changed with repeated samples; subjects with at least 4 samples were included in this analysis. In addition, this same group of patients was used to study the association between antibody levels, positivity, number of positive markers and quartile group with Crohn's disease complications; Wilcoxon rank sum tests and Pearson's chi-square tests were used, as appropriate. A p<0.05 was considered statistically significant, unless otherwise stated. SAS version 9.2 software (The SAS Institute, Cary, NC) and R version 2.10.1 (The R Foundation for Statistical Computing, Vienna, Austria) were used for all analyses.

## Results

20% of the sera were collected within six months, 30% within one year of diagnosis. The frequency of testing for the individual patients can be seen in **Table 1**. The median time between sample procurements in the subjects was 6.2 months (Interquartile range (IQR) 3.5 months–12.2 months).

### I) Definition of level and status changes over time

As not all samples were taken at time of diagnosis and follow-up times varied we first tested if these two criteria have an influence on our analysis. The magnitude of level changes in our IBD cohort was not dependent on the time from diagnosis to first sample procurement. In addition, there was no significant time-level association between subjects with a short or long follow-up period, indicating that the observed level changes are independent of the observation time (data not shown). This suggests that our cohort can be used for an analysis of fluctuations over time, despite not all samples being taken at the time of IBD diagnosis or with a fixed follow-up time.

#### Level changes in Crohn's disease

**Figure 1:**
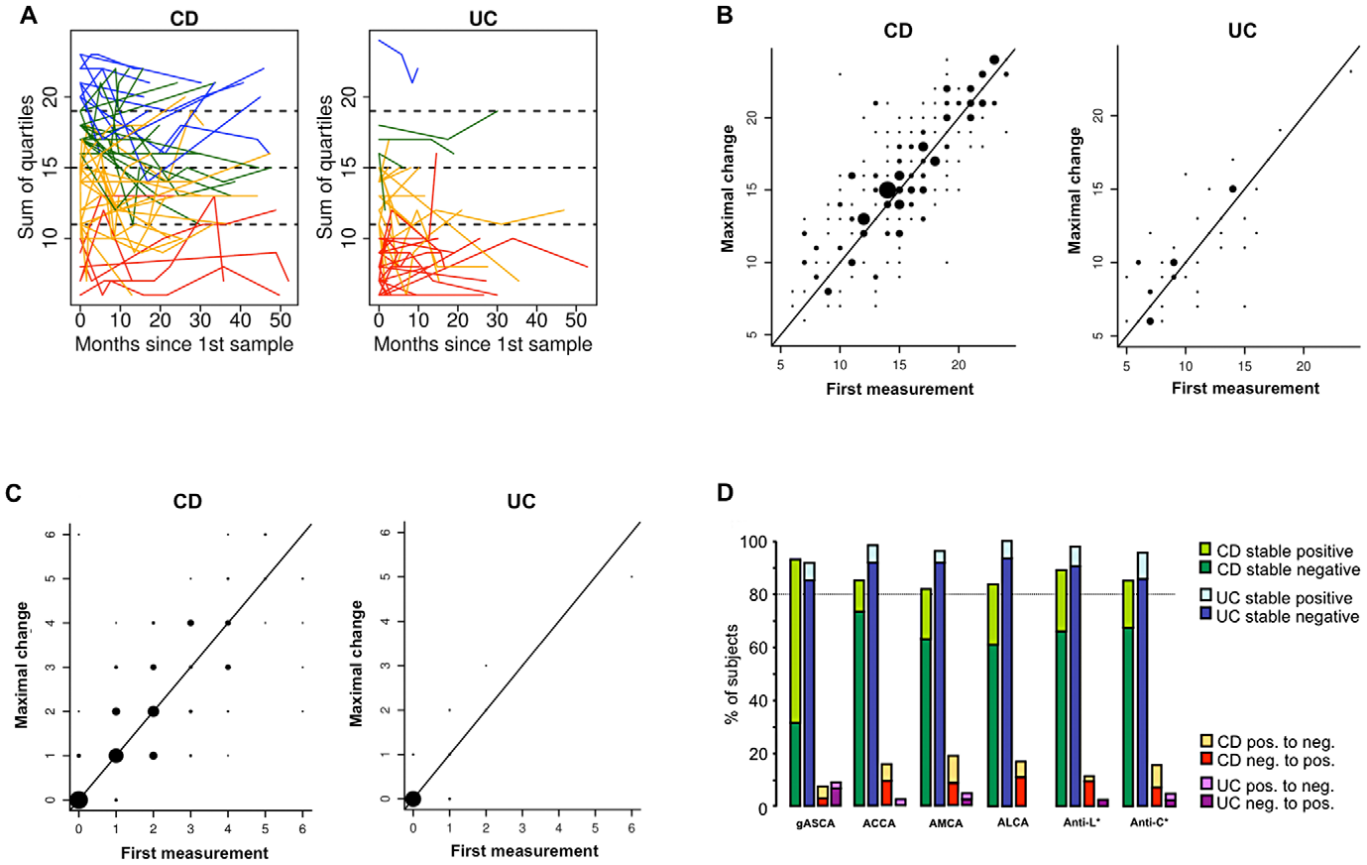
A. Changes in quartile sum score over time. Profile plots for changes in the quartile sum score (QSS) in individual Crohn's disease (CD) and Ulcerative colitis (UC) patients over time. The broken lines represent four equal sections of the QSS. The blue, green, yellow and red lines indicate patients starting in a certain section of the QSS. Depicted is a random set of 50 subjects per graph. B. Maximal changes in the quartile sum score. Scatter plots comparing the quartile sum score (QSS) of the first sample and the sample with the maximal changes in QSS during follow-up in Crohn's disease (CD) and Ulcerative colitis (UC) patients. The size of the dots represents the number of patients per datapoint. C. Maximal changes in number of positive markers. Scatter plots comparing the number of positive markers of the first sample and the sample with the maximal changes in quartile sum score during follow-up in Crohn's disease (CD) and Ulcerative colitis (UC) patients. The size of the dots represents the number of patients per datapoint. D. Stability in antibody status over time. First sample compared to sample with with the maximal changes in quartile sum score during follow-up in Crohn's disease (CD) and Ulcerative colitis (UC) patients. Values are presented as N (%) for changes in marker status. gASCA: anti-*Saccharomyces cerevisiae* antibodies, ACCA: anti-chitobioside carbohydrate IgA antibodies, ALCA: anti-laminaribioside carbohydrate IgG antibodies, AMCA: anti-mannobioside carbohydrate IgG antibodies, Anti-L: anti-laminarin carbohydrate antibody, Anti-C: anti-chitin carbohydrate antibody; pos.: positive, neg.: negative; *Anti-L and Anti-C were not available in all patients.

We next characterized the level changes of the glycan markers in patients with CD. We noted substantial changes of the overall immune response depicted by the QSS and of levels of all tested single serum markers in a subgroup of individual CD patients over time (**Figure 1** showing QSS for CD; **Figure S1** showing the levels of the individual markers in CD patients). To describe the magnitude of level changes in CD patients we first divided the QSS into four equal sections, assigned the CD subjects to a respective section and identified patients with changes of their QSS section over time (color coded in **Figure 1A**). Between 47.1% and 79% of the CD patients per section (versus 0% to 50% of the UC subjects; **Figure 1A**) changed their QSS section at least once during follow-up.

We next categorized the *average* fluctuation per individual patient by calculating the Z-scores (level changes in standard deviations (SD) from the per-patient mean) for all patients and samples. When taking into account the average Z-scores per patient, which includes all observed marker changes, we found while the absolute changes of the marker levels are more pronounced in CD versus UC the relative changes are comparable, which is true for the QSS and the levels of all single markers (**Table S1**).

To classify the *maximal* extent of level changes in individual CD patients over time we identified the samples with the maximal change in QSS (increase or decrease) compared to the first available sample per individual CD patient during follow-up. In other words we identified the two samples per individual patient with the maximal changes in serologic immune response to the tested glycan epitopes. The comparison of the first sample and the sample with the maximal QSS changes during follow-up can be seen in **Figure 1B**. We also assessed the *maximal* level changes for each individual antibody (**Table 2** **; Figure S2**). This further corroborated the finding of marked fluctuations in the overall immune response in a subgroup of CD patients. In CD patients the maximal Z-score per patient was 0.91 (SD 0.38) (versus UC 0.76 (SD 0.36)) again indicating that the relative level changes are comparable between CD and UC.

**Table 2:** Changes in antibody levels: First sample compared to sample with maximal change in sum of quartiles.

Marker	CD(N = 207)	UC(N = 46)
**Absolute changes in levels, median (EU (P25, P75))**		
gASCA	10.7 (4.1, 25.1)	2.8 (1.2, 9.6)
ACCA	14.4 (7.1, 25.8)	9.1 (3.3, 17.0)
AMCA	23.5 (8.8, 41.8)	11.8 (7.9, 21.9)
ALCA	8.5 (3.0, 16.6)	4.5 (1.8, 6.8)
Anti-L	21.5 (9.3, 41.1)	8.9 (5.3, 14.9)
Anti-C	8.0 (3.6, 16.8)	6.3 (3.3, 14.7)
Sum of quartiles	2.0 (1.0, 3.0)	1.0 (1.0, 3.0)
**Absolute % changes from baseline (Median (P25, P75))**		
gASCA	19.0 (6.1, 33.1)	17.7 (6.8, 35.3)
ACCA	26.7 (14.8, 45.5)	19.5 (7.1, 37.0)
AMCA	31.6 (13.7, 50.1)	21.2 (15.1, 39.0)
ALCA	21.8 (8.5, 39.8)	17.0 (7.7, 31.7)
Anti-L	26.7 (12.0, 57.2)	18.3 (12.3, 29.4)
Anti-C	24.5 (11.3, 39.2)	27.6 (13.8, 39.3)
Sum of quartiles	11.1 (5.6, 23.1)	13.8 (7.1, 25.0)

gASCA: anti-*Saccharomyces cerevisiae* antibodies, ACCA: anti-chitobioside carbohydrate IgA antibodies, ALCA: anti-laminaribioside carbohydrate IgG antibodies, AMCA: anti-mannobioside carbohydrate IgG antibodies, Anti-L: anti-laminarin carbohydrate antibody, Anti-C: anti-chitin carbohydrate antibody.

CD: Crohn's disease; UC: Ulcerative colitis; EU: ELISA units; P25, P75: 25th and 75th percentiles.

Taken together we show that on average across the whole CD cohort individual marker levels are rather stable, with however certain patients exhibiting stronger *average* and *maximal* fluctuations. The relative level changes are comparable in CD versus UC, with stronger absolute changes in CD.

#### Status changes in Crohn's disease

A different way to assess stability in serologic markers is to use a dichotomic categorization - positive or negative for a respective antibody or if investigating a panel the number of positive markers. We therefore tested how often the levels of the single markers cross the respective cut-off values over time, changing their status from positive to negative or vice versa. In CD subjects in a median of 3.3% of the visits (IQR 0.0–11.1; minimum (min.) 0, maximum (max.) 50) any of the marker status changed compared to the previous consultation (versus UC with a median of 0% of the visits (IQR 0; min. 0, max. 16.7).

Considering the two samples per individual patient with the *maximal* changes in immune response during follow-up we next characterized the longitudinal CD patient samples for stability in marker status (positive versus negative for a respective antibody). We found a strikingly stable antibody status for both the total number of positive markers over time (**Figure 1C**) as well as for each single antibody (**Figure 1D**) per individual patient. Only between 7.3% (ASCA) and 18.3% (AMCA) of the CD patients changed the antibody status of an individual marker either from positive to negative or from negative to positive. This indicates while marked level changes occur in a subgroup of CD patients the antibody status is largely stable.

#### Level and status changes in ulcerative colitis

As partly stated above and in contrast to the serum of CD patients the absolute changes in the overall immune response and in the antibody status over time in UC patients were minute (**Table 2** **;** **Figure 1** **; Figure S3**). Only between 0% and 8.7% of the UC patients changed the antibody status of an individual marker during follow-up.

### II) Association of level or status changes with clinical phenotypes

After defining the magnitude of level changes over time we aimed to assess clinical and genetic factors that are associated with stronger versus weaker fluctuations in the serologic markers. This information is critical for identifying the subgroup of patients prone to more pronounced changes in their antibody levels during the disease course. As the absolute antibody levels and status in UC patients were highly stable over time we continued our analysis with the CD cohort only. At time of first sample procurement neither disease activity nor an elevated CRP were linked to increased or decreased QSS or levels or status of single markers.

We first tested the *average* fluctuation per individual patient using all samples over time. For this purpose a Z-score was calculated for each visit, averaged and associated with the above clinical phenotypes or NOD genotypes (**Tables S2 & S3**). We corrected for follow-up time and used a level of significance of p<0.01 to account for multiple testing. No phenotype or NOD2 genotype was associated with a higher magnitude of *average* fluctuations in the marker levels. The maximal difference in Z-score between the groups was 0.06 SD and cannot be considered clinically relevant.

To identify factors associated with *maximal* level fluctuations we divided our CD patients in two different groups based on the magnitude of *maximal* level changes in individual patients over time: “Non-level changers” (stable QSS over time, remaining within 0.5 standard deviations (SD) of the QSS in the initial sample) and “Any level changers” (increase or decrease >0.5 SD in QSS at any time during follow-up). These two groups were analyzed for association with certain clinical disease phenotypes and NOD2 genotypes (**Table S4**). To control for multiple testing we used a significance level of p<0.01 and the results were adjusted for follow-up time. We did not find any association between the magnitude of *maximal* level fluctuation in our cohort and any of the pheno- or NOD2 genotypes. We performed this analysis with considering only CD-patients with a minimal observation period of 12 months. Also here no association of pheno- or genotypes with fluctuations was found (data not shown).

The antibody levels in the first sample did not predict the degree of fluctuation of the markers at later time points in the disease course as revealed by a logistic regression analysis (data not shown). In other words the same degree of maximal level changes occurred in subjects during follow-up that had high, middle or low levels in the first sample. We additionally separated our cohort into two groups according to changes in marker status: “Non-status changers” (patients that did not change the number of positive markers during follow-up) and “Status changers” (patients that became positive or negative for one or more additional markers during follow-up). The distribution of subjects can be found in **Table S5**. We also assessed the groups for association with certain clinical disease phenotypes and NOD2 genotypes (**Table S6**). We accounted for multiple testing by using a significance level of p<0.01 and adjusted for follow-up time. No association of pheno- or genoptypes with fluctuations was found, when considering the whole cohort but also when separately analyzing only CD patients with a minimum follow-up of 12 months (data not shown).

In summary, despite considering a broad range of clinical phenotypes and NOD2 genotypes no associations with *average* or *maximal* changes in marker levels and status over time could be found.

### III) Level changes in individual patients over time – a longitudinal analysis

**Figure 2:**
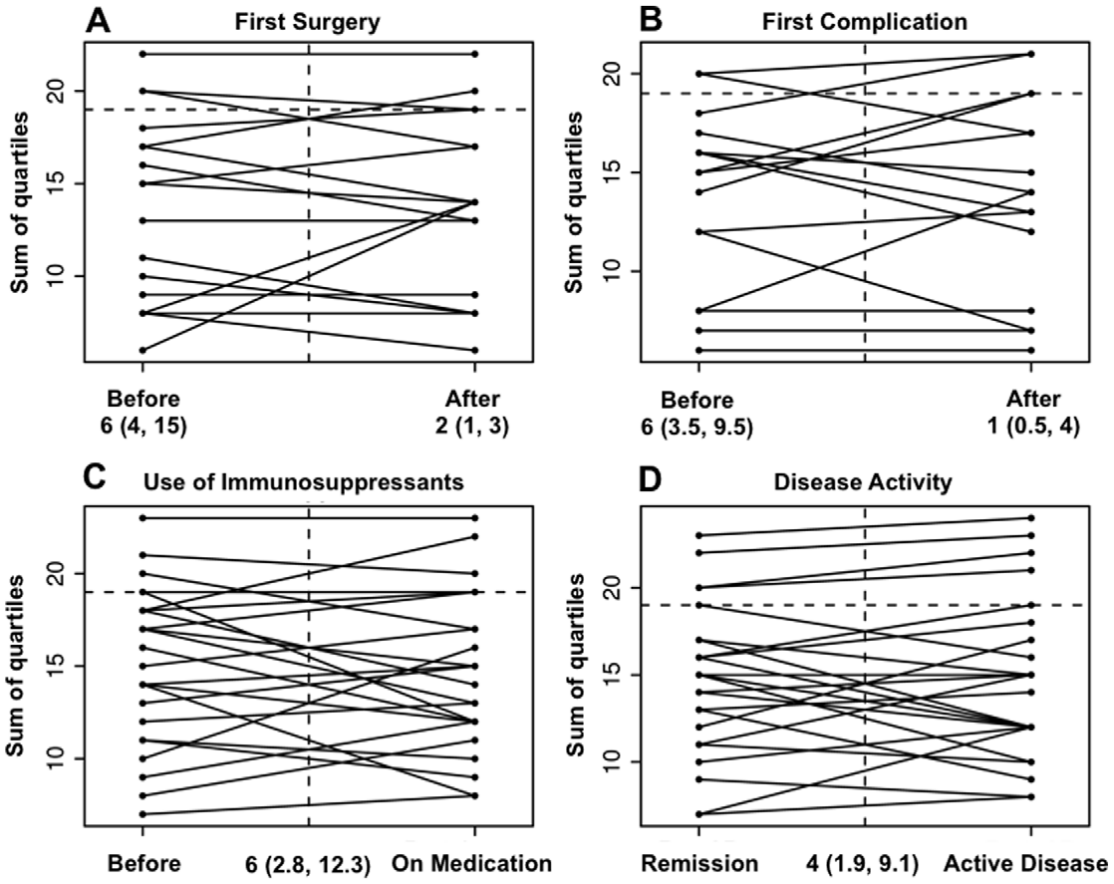
Change in the serum immune response in relation to clinical events. Change in antibody levels (quartile sum score) with (A) the first occurrence of Crohn's disease (CD)-related surgery (n = 17), (B) complications (fistulae or strictures; n = 16), (C) new intake of immunsuppressive medication (depicted is a random set of 25 patients out of total n = 152) or (D) onset of active disease (depicted is a random set of 25 patients out of total n = 124). Numbers relate to months before or after the event for A & B and months between the two samples for C & D (interquartile range (IQR)).

Even though a certain clinical phenotype or NOD2 genotype was not associated with stronger fluctuations *per se*, it is well likely that changes in CD behavior or treatment can lead to changes in the levels of the antibody markers over time. We therefore longitudinally analyzed our CD cohort for potential level changes inflicted by clinical situations, namely the first occurrence of complicated CD-behavior, the first occurrence of CD-related surgery, the use of immunosuppressive medication or the onset of active disease. For this purpose repeated measures analysis was done using an unstructured covariance matrix to model within-subject correlation. The occurrence of first time complicated CD-behavior, first time CD-related surgery, active disease or the start of immunosuppressive medication did not influence the overall immune response or the levels of the individual markers. Pre-event antibody levels were not significantly different to post-event levels taking into consideration the above parameters (**Table 3** and **Figure 2** for the QSS; **Table S7** for the single markers). When looking at the use of corticosteroids separately we found a trend towards a decrease in the QSS (data not shown). Interestingly, overall the QSS mildly increased over time from diagnosis (0.13 points each year), which was due to a level increase in the IgA marker ACCA with a trend to increased levels for Anti-L and Anti-C (**Table 3**). This indicates a good model fit as this is a known phenomenon for antibodies of the IgA class.

**Table 3:** Longitudinal analysis of level changes inflicted by clinical situations.

	Sum of quartiles	
Factor	Parameter Estimate(SE)	p-value
Months since diagnosis	0.011 (0.003)	***0.002***
Change in disease activity	−0.347 (0.443)	0.43
First occurrence of complicated CD behavior	0.021 (0.408)	0.96
First occurrence of CD-related surgery	−0.654 (0.999)	0.51
Use of immunosuppressants	0.410 (0.871)	0.64

SE: Standard error; CD: Crohn's disease.

### IV) Clinical situations over time – a longitudinal analysis

The most important factor for the performance of the marker panel is, if the accuracy in clinical situations remains unchanged over time. We therefore tested, if the observed fluctuations in marker levels and status alter the capability of the glycan marker panel to differentiate between CD versus UC and their association with CD phenotypes. We used serum of the CD patients where at least four different samples were available during follow-up (n = 75). The expression of the glycan markers, the clinical phenotype and the overall immune response in this subpopulation was comparable to the total CD cohort (n = 207), indicating that no selection bias was present.

We assessed changes in the ability of the markers to differentiate CD *versus* UC (**Table 4**). We did not observe any significant changes in discriminatory capacity of the markers over time. We next investigated potential changes in association with CD phenotypes. 87.7% of the CD patients with at least four available samples already had a complication before sample procurement at first sample procurement, a proportion that increased to 90.8% (2^nd^ sample), and 93.3% (3^rd^ and 4^th^ sample) during follow-up. 85% of the CD patients already underwent surgery once at time of first sample, which increased during follow-up to 90% for the other three time points. Also here an overall stable association pattern between the markers and clinical phenotypes was found. Independent of the time of sample procurement over the follow-up period the overall immune response, as depicted by the sum of quartiles or the number of positive markers remained to be associated with ileal involvement, complicated CD behavior or CD-related surgery (**Table 5** **; Table S8** for all single markers).

**Table 4:** Validity of markers for differentiation of CD versus UC over time in individual patients.

Antibody	Sensitivity (%)	Specificity (%)	PPV (%)	NPV (%)
***1st Sample***				
gASCA	69.3	91.3	92.9	64.6
ACCA	24	91.3	81.8	42.4
AMCA	29.3	93.5	88	44.8
ALCA	34.7	95.7	92.9	47.3
Anti-L	28.4	93	87.5	43
Anti-C	36.5	88.4	84.4	44.7
***2nd Sample***				
gASCA	69.3	91.3	92.9	64.6
ACCA	17.3	91.3	76.5	40.4
AMCA	24	93.5	85.7	43
ALCA	29.3	95.7	91.7	45.4
Anti-L	28.4	93	87.5	43
Anti-C	25.7	88.4	79.2	40.9
***3rd Sample***				
gASCA	69.3	91.3	92.9	64.6
ACCA	18.7	91.3	77.8	40.8
AMCA	21.3	93.5	84.2	42.2
ALCA	33.3	95.7	92.6	46.8
Anti-L	33.3	93	89.3	44.4
Anti-C	20	88.4	75	38.8
***4th Sample***				
gASCA	70.7	91.3	93	65.6
ACCA	10.7	91.3	66.7	38.5
AMCA	17.3	93.5	81.3	41
ALCA	30.7	95.7	92	45.8
Anti-L	31.1	93	88.5	44
Anti-C	31.1	88.4	82.1	42.7

Includes 75 CD subjects with at least four samples per individual patient versus all 46 UC subjects.

PPV: positive predictive value, NPV: negative predictive value.

gASCA: anti-*Saccharomyces cerevisiae* antibodies, ACCA: anti-chitobioside carbohydrate IgA antibodies, ALCA: anti-laminaribioside carbohydrate IgG antibodies, AMCA: anti-mannobioside carbohydrate IgG antibodies, Anti-L: anti-laminarin carbohydrate antibody, Anti-C: anti-chitin carbohydrate antibody.

**Table 5:** Validity of markers for association with disease phenotypes over time in individual patients.

	Ileum Involvement	No Ileum Involvement		Complication	No Complication		Surgery	No Surgery	
Factor	(N = 67)	(N = 8)	p-value	(N = 65)	(N = 10)	p-value	(N = 60)	(N = 15)	p-value
***1st Sample***									
**Sum of quartiles**	16.0 (13.5, 19.5)	10.0 (8.0, 14.0)	***<0.001***	17.0 (13.0, 20.0)	10.0 (8.0, 14.0)	***<0.001***	17.5 (14.0, 21.0)	14.0 (10.0, 16.0)	***0.003***
**Number of positive markers**	2.0 (1.0, 3.0)	0.0 (0.0, 1.0)	***<0.001***	2.0 (1.0, 4.0)	0.0 (0.0, 1.0)	***<0.001***	2.0 (1.0, 4.0)	1.0 (0.0, 2.0)	***0.002***
***2nd Sample***									
**Sum of quartiles**	15.5 (12.0, 19.0)	10.0 (8.0, 14.0)	***<0.001***	16.0 (12.0, 19.0)	10.0 (8.0, 13.0)	***<0.001***	16.5 (13.0, 19.5)	12.0 (12.0, 15.0)	***0.004***
**Number of positive markers**	2.0 (1.0, 3.0)	0.0 (0.0, 1.0)	***<0.001***	2.0 (1.0, 3.0)	0.0 (0.0, 1.0)	***<0.001***	2.0 (1.0, 3.0)	1.0 (0.0, 2.0)	***0.003***
***3rd Sample***									
**Sum of quartiles**	15.0 (12.0, 19.0)	10.0 (8.0, 14.0)	***<0.001***	15.0 (12.0, 19.0)	9.0 (7.0, 13.0)	***<0.001***	16.5 (13.0, 19.0)	11.0 (8.0, 15.0)	***0.004***
**Number of positive markers**	2.0 (1.0, 3.0)	0.0 (0.0, 1.0)	***<0.001***	2.0 (1.0, 3.0)	0.0 (0.0, 1.0)	***<0.001***	2.0 (1.0, 3.0)	1.0 (0.0, 2.0)	***0.006***
***4th Sample***									
**Sum of quartiles**	15.0 (12.0, 19.0)	10.0 (8.0, 14.0)	***<0.001***	15.0 (12.0, 19.0)	10.0 (8.0, 14.0)	***<0.001***	15.0 (13.0, 20.0)	13.0 (10.0, 16.0)	***0.02***
**Number of positive markers**	2.0 (1.0, 3.0)	0.0 (0.0, 1.0)	***<0.001***	2.0 (1.0, 3.0)	0.0 (0.0, 1.0)	***<0.001***	2.0 (1.0, 3.0)	1.0 (0.0, 2.0)	***0.025***

Includes 75 CD subjects with at least four samples per individual patient.

Sum of quartiles: Values presented are median (25th percentile, 75th percentile) and P-values correspond to Wilcoxon rank sum tests.

Antibody positivity: Values presented are N (%) and P-values correspond to Fisher's Exact test (F) and Pearson's chi-square tests otherwise.

Complication: fistula or stenosis.

## Discussion

There is a growing interest in the use of anti-glycan antibodies as markers for differentiation and stratification of CD. Previous reports, including ours [7], [8], [9], [10], [11], [13], suggest a potential use of these markers for discrimination between CD and UC as well as prediction of complicated CD behavior and CD-related surgery. Most information, however, is derived from cross-sectional studies [7], [8], [9], [11], [13], which should be interpreted with caution, because crucial information is missing about the stability of marker levels and status over time in individual patients, an open question we specifically addressed in our investigation.

We detected marked changes in the overall immune response (QSS) and levels of individual markers in a subgroup of CD subjects over time. The marker status (positive versus negative) remained widely stable. None of our tested clinical phenotypes or NOD2 genotypes was associated with stronger *average* or *maximal* changes in marker levels. In a longitudinal analysis with individual patients over time neither changes in disease activity, CD behavior or surgery, nor the intake of immunosuppressive medication led to changes in the QSS. The ability of the panel to discriminate CD *versus* UC or its association with CD location, behavior or surgery remained stable during the follow-up time. In UC neither significant absolute level nor status changes were observed.

Incomplete and limited information is available on fluctuations in level and status of serum antibodies directed against microbial components and associated with CD over time. Most data derived from *cross-sectional single point studies* are controversial: In 32% of apparently healthy recruits of the Israeli Defence Forces, who were later diagnosed as having CD, levels of ASCA increased closer to the date of diagnosis [27]. Once diagnosis of CD was established disease duration did not seem to influence serological responses in several reports, investigating ASCA, OmpC, Anti-I2 and CBir1 [19], [21]. In contrast to this, others found significant higher antibody responses against ASCA, AMCA, ACCA and OmpC associated with increased disease duration [7], [9]. Oshitani et al. demonstrated lower ASCA titers in CD patients taking mesalazine compared to CD patients not taking mesalazine [17]. In contrary, Ruemmele et al. reported no difference in ASCA positivity with steroid and mesalazine treatment in a pediatric population. A decline in ASCA levels towards normal values had been observed post-surgically in CD [16]. Changes in disease activity did not seem to influence marker levels [9], [11].

The information derived from *longitudinal studies* in individual patients over time is even scarcer and meaningful conclusions are difficult to make due to limited follow-up times or low patient numbers. Dotan et al. followed the serum levels of the glycan-markers anti-alpha-Rha, anti-alpha-GlcNAc and anti-cellotriose antibodies in seven healthy volunteers during the course of 13 weeks and found widely stable levels over time [28]. In a pediatric cohort (n = 61) with a mean follow-up time of 4.9 years and up to seven measurements during the CD course only 21 to 29.5% of the subjects changed ASCA status over time. Even though individual patients showed marked changes in titers the average levels across the total population showed little variability during follow-up [23].

Disease activity or relapse does not appear to influence marker levels in longitudinal studies [23], [29]. ASCA was stable over time in relation to changes in CDAI, monitoring 26 CD patients before and after infliximab therapy for a median interval of 6.1 months [19]. Among patients who achieved complete remission through treatment with infliximab as evidenced by mucosal changes and healing, stability in anti-CBir1 expression was seen (n = 14) [21]. ASCA titers in CD patients taking mesalazine *versus* placebo for prophylaxis of postoperative relapse (n = 38) remained stable over time [20]. However, ASCA levels decreased after two weeks of corticosteroid treatment (n = 25, follow-up 9 weeks) [20]. A decrease in ASCA levels after intestinal surgery with a later rise to preoperative levels within a few months was noted (n = 60) [22]. In contrast no significant changes in serological responses toward ASCA, Anti-I2 and Omp-C after small bowel surgery (n = 61 and 26) with or without fecal diversion (n = 14) was found [14], [23], [24].

Our study describes the dynamic change of anti-glycan antibodies in the to date largest cohort of CD patients with multiple samples collected during the disease course and scrutinizes detailed clinical information for each time point of sample procurement. Comparable studies but of smaller size have so far only been reported for ASCA [14], [22], [23].

We established that during the disease course significant fluctuations in marker levels occur in a subgroup of CD patients. The marker status, however, remained strikingly stable. Considering all markers a change in status occurred in only 3.3% of the visits per antibody. In an attempt to identify specific clinical phenotypes or genotypes that are associated with stronger level or status changes we included a wide range of phenotypes into our statistical analysis. Interestingly none of the clinical phenotypes was associated with level or status changes in antibodies over time. The immune response tended to decrease with intake of corticosteroids. This is in concordance with a previous report [20], but the effect was not noted when all immunosuppressive medications (including corticosteroids, azathioprine and methotrexate) were considered in the model. None of our patients received infliximab during the observation period.

The fact that our association analysis for disease phenotypes confirms the previously reported results from our larger cohort [11] underlines the appropriate power of our study to detect potential changes in marker levels and status, if they were present. In addition our longitudinal analysis detected a mild increase in the levels of the glycan IgA markers over time indicating a good model fit, because a rise in the serum titers of IgA antibodies over time is a known physiologic phenomenon. We see two possible explanations for our lack of association of phenotypes/phenotype changes with fluctuations: A clinical phenotype or genotype not considered in our model might lead to the observed changes. Alternatively our observation may reflect the natural fluctuation of the markers/immune response over time. This is supported by the finding of comparable results in *relative* changes of the marker levels (**Table 2**) with essentially identical Z-scores between CD and UC. UC is not associated with higher levels of anti glycan antibodies and, therefore, the absolute fluctuations are minute and not clinically relevant. The onset of level changes inflicted by clinical situation might be delayed, e.g. an increase in serologic glycan marker levels might lag behind an increase in disease activity and therefore would not be detected in our longitudinal analysis.

Determination of glycan markers in clinical practice may allow the differentiation between UC and CD, which could impact the type of therapy chosen for the respective disease. In addition, the ability to predict the progression from non-complicated to complicated disease and/or surgery with serum markers may classify patients into at-risk populations and influence the therapeutic management of patients with the goal of preventing the development of complications. One prerequisite for routine use is the stability of markers over time. The most important finding of our study is that despite the observed changes in the QSS the potency of the markers in clinical situations, namely discrimination between UC versus CD and association with CD phenotypes remained stable over time. Given the marker status stability over time in our study, association of marker positivity with CD and certain disease phenotypes are likely to be suitable as a tool for differentiation and prediction of disease courses, independent of the time of sample procurement. Therefore, serial measurements of antibodies may not provide additional information for the evaluation of CD when considering the status of the markers. On the other hand the marked fluctuations in the QSS in a subgroup of CD patients suggests caution when using the overall immune response for the aforementioned clinical situations, namely diagnosis, differential diagnosis and disease stratification.

The strength of our study is its originality by being the first study investigating fluctuations in anti-glycan antibodies, the prospective follow-up design, blinded data abstraction, availability of up to 11 samples per patient and a longitudinal analysis paired with detailed clinical information. However, certain limitations apply: One has to keep in mind that our cohort is from a single university hospital, introducing possible referral bias. The first serum sample per patient was not in all cases taken close to diagnosis, which we corrected for in our statistical models. The follow-up samples were taken at arbitrary visits to our hospital and not in a fixed relation to certain events such as complications or surgery, information that was retrospectively added. Using this method, patients with a more severe disease course could be selected out, as only they have to come to a referral center for multiple treatments. The length of time in which immunosuppressive medication was taken by the patients before sample procurement is unknown to the authors, which is a limitation for our longitudinal model. We are aware of the potential presence of a subclinical complication at the time of sample procurement. One has to consider that this study does not aim at prediction of complicated CD courses with serology, but rather evaluates the fluctuations of the glycan markers longitudinally to assess, if and how already published cross sectional studies can be used for disease prediction. Even though this is the largest study of its kind ever reported, the overall number of patients is limited. A larger cohort would enable a more detailed analysis with respect to determinants of level changes.

In summary, our study indicates that anti-glycan antibody levels are changing in patients with CD whereas the status of the markers is stable over time. The observed fluctuations might be due to not yet identified clinical factors or genotypes or could represent natural changes in levels over time. Considering the follow-up time of our study we cannot recommend serial measurements, when considering the marker status and claim to use the overall immune response (QSS) with caution for disease stratification, due to strong fluctuations in a subgroup of CD subjects.

## Supporting Information

Figure S1 **Changes in the level of single markers over time.** Profile plots for changes in the levels of single markers in individual Crohn's disease (CD) patients over time. The broken line represents the cut-off value for each individual marker. The red lines indicate patients starting above the cut-off value and the green lines indicate subjects starting below the cut-off values. Depicted is a random set of 50 subjects per graph. gASCA: anti-*Saccharomyces cerevisiae* antibodies, ACCA: anti-chitobioside carbohydrate IgA antibodies, ALCA: anti-laminaribioside carbohydrate IgG antibodies, AMCA: anti-mannobioside carbohydrate IgG antibodies, Anti-L: anti-laminarin carbohydrate antibody, Anti-C: anti-chitin carbohydrate antibody.
(TIF)

Figure S2 **Maximal changes in levels of single markers in Crohn's disease.** Scatter plot comparing the level of the first sample and the sample with the maximal changes in quartile sum score during follow-up for each individual marker in Crohn's disease (CD) patients. One dot represents one patient. gASCA: anti-*Saccharomyces cerevisiae* antibodies, ACCA: anti-chitobioside carbohydrate IgA antibodies, ALCA: anti-laminaribioside carbohydrate IgG antibodies, AMCA: anti-mannobioside carbohydrate IgG antibodies, Anti-L: anti-laminarin carbohydrate antibody, Anti-C: anti-chitin carbohydrate antibody.
(TIF)

Figure S3 **Maximal changes in levels of single markers in Ulcerative colitis.** Scatter plot comparing the level of the first sample and the sample with the maximal changes in quartile sum score during follow-up for each individual marker in Ulcerative colitis (UC) subjects. One dot represents one patient. gASCA: anti-*Saccharomyces cerevisiae* antibodies, ACCA: anti-chitobioside carbohydrate IgA antibodies, ALCA: anti-laminaribioside carbohydrate IgG antibodies, AMCA: anti-mannobioside carbohydrate IgG antibodies, Anti-L: anti-laminarin carbohydrate antibody, Anti-C: anti-chitin carbohydrate antibody.
(TIF)

Table S1 Average of absolute standard deviations around the mean (Z-score).
(TIFF)

Table S2 Average of absolute standard deviations around the mean (Z-score) and association with disease pheno- and NOD2 genotypes.
(TIFF)

Table S3 Correlation coefficients of the Z-score with disease parameters.
(TIFF)

Table S4 Association of clinical phenotypes and genotypes with maximal level changes.
(TIFF)

Table S5 Number and distribution of CD subjects with antibody status changes.
(TIFF)

Table S6 Assosiation of clinical phenotypes and genotypes with status changes.
(TIFF)

Table S7 Longitudinal analysis of level changes inflicted by clinical situations.
(TIFF)

Table S8 Validity of markers for association with disease phenotypes over time in individual patients.
(TIFF)
