# Supplementary figures and images for: Characterization of Changes in Serum Anti-Glycan Antibodies in Crohn's Disease – a Longitudinal Analysis
Source: PLoS One. 2011 May 6;6(5):e18172. doi: 10.1371/journal.pone.0018172 (PMC3089599; doi:10.1371/journal.pone.0018172)

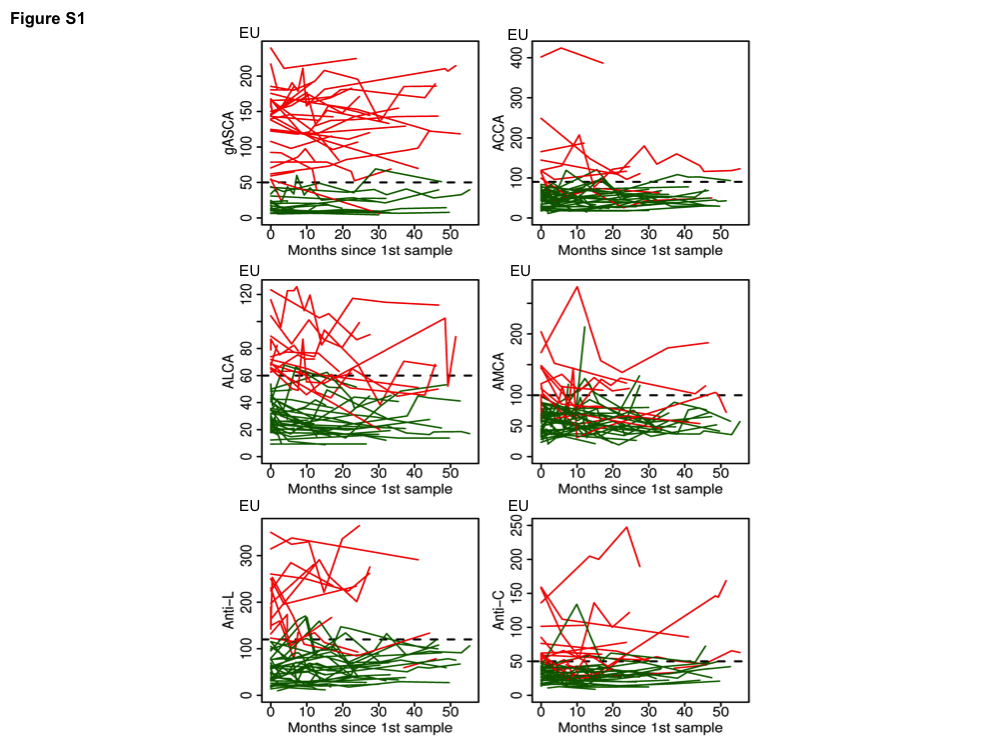

Supplement: Figure S1 — Changes in the level of single markers over time. Profile plots for changes in the levels of single markers in individual Crohn's disease (CD) patients over time. The broken line represents the cut-off value for each individual marker. The red lines indicate patients starting above the cut-off value and the green lines indicate subjects starting below the cut-off values. Depicted is a random set of 50 subjects per graph. gASCA: anti-Saccharomyces cerevisiae antibodies, ACCA: anti-chitobioside carbohydrate IgA antibodies, ALCA: anti-laminaribioside carbohydrate IgG antibodies, AMCA: anti-mannobioside carbohydrate IgG antibodies, Anti-L: anti-laminarin carbohydrate antibody, Anti-C: anti-chitin carbohydrate antibody. (TIF) [file pone.0018172.s001.tif]

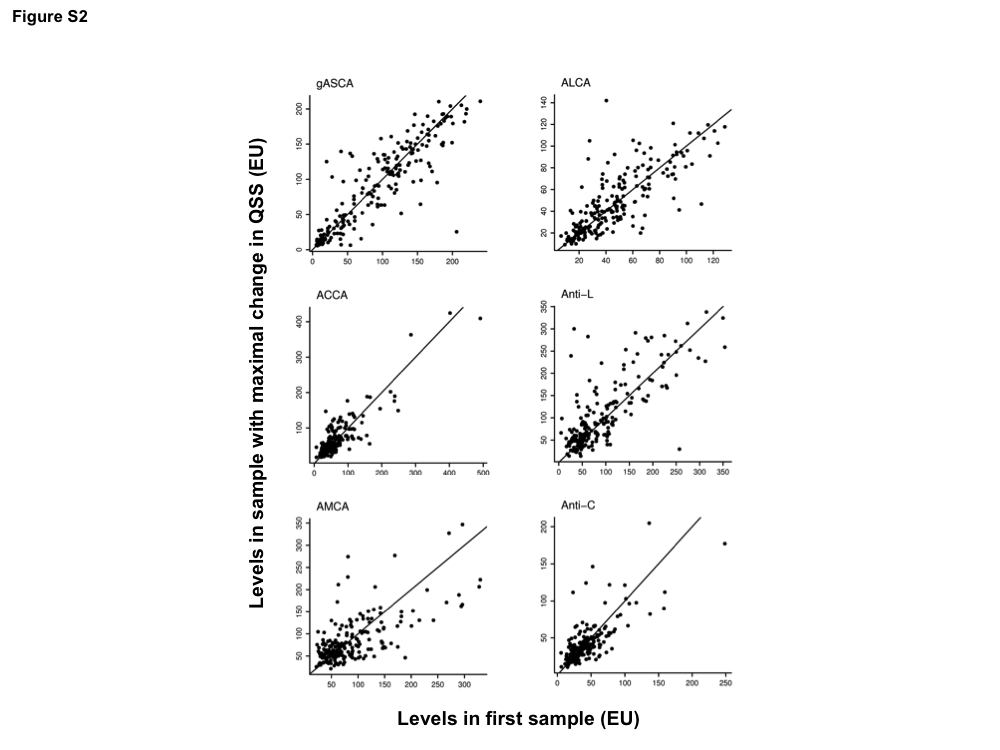

Supplement: Figure S2 — Maximal changes in levels of single markers in Crohn's disease. Scatter plot comparing the level of the first sample and the sample with the maximal changes in quartile sum score during follow-up for each individual marker in Crohn's disease (CD) patients. One dot represents one patient. gASCA: anti-Saccharomyces cerevisiae antibodies, ACCA: anti-chitobioside carbohydrate IgA antibodies, ALCA: anti-laminaribioside carbohydrate IgG antibodies, AMCA: anti-mannobioside carbohydrate IgG antibodies, Anti-L: anti-laminarin carbohydrate antibody, Anti-C: anti-chitin carbohydrate antibody. (TIF) [file pone.0018172.s002.tif]

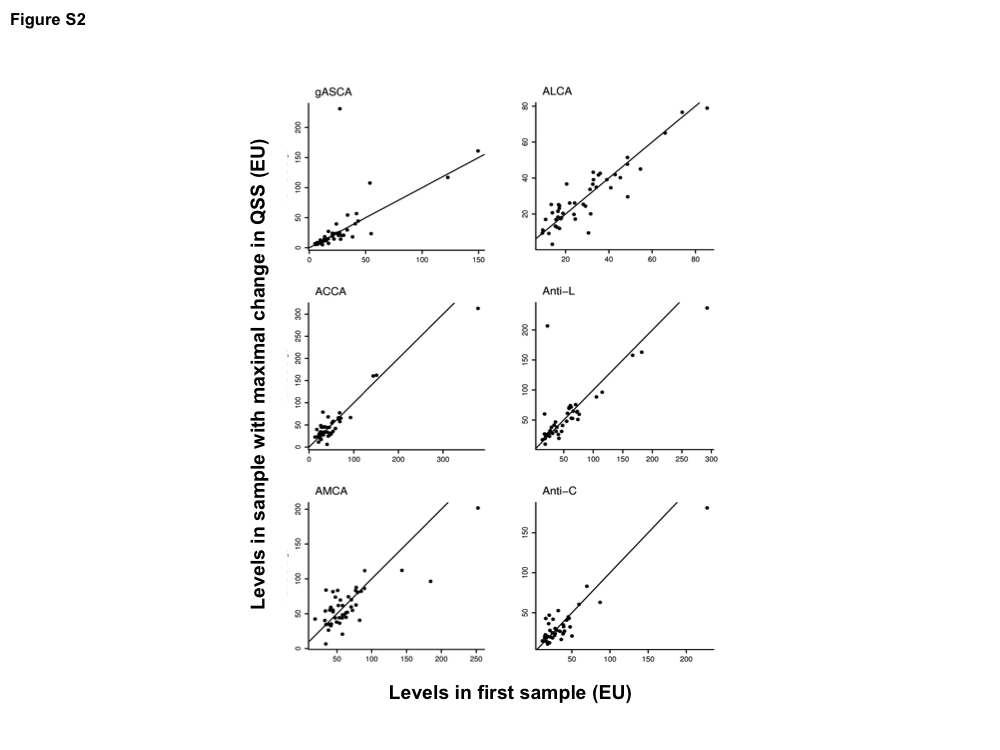

Supplement: Figure S3 — Maximal changes in levels of single markers in Ulcerative colitis. Scatter plot comparing the level of the first sample and the sample with the maximal changes in quartile sum score during follow-up for each individual marker in Ulcerative colitis (UC) subjects. One dot represents one patient. gASCA: anti-Saccharomyces cerevisiae antibodies, ACCA: anti-chitobioside carbohydrate IgA antibodies, ALCA: anti-laminaribioside carbohydrate IgG antibodies, AMCA: anti-mannobioside carbohydrate IgG antibodies, Anti-L: anti-laminarin carbohydrate antibody, Anti-C: anti-chitin carbohydrate antibody. (TIF) [file pone.0018172.s003.tif]

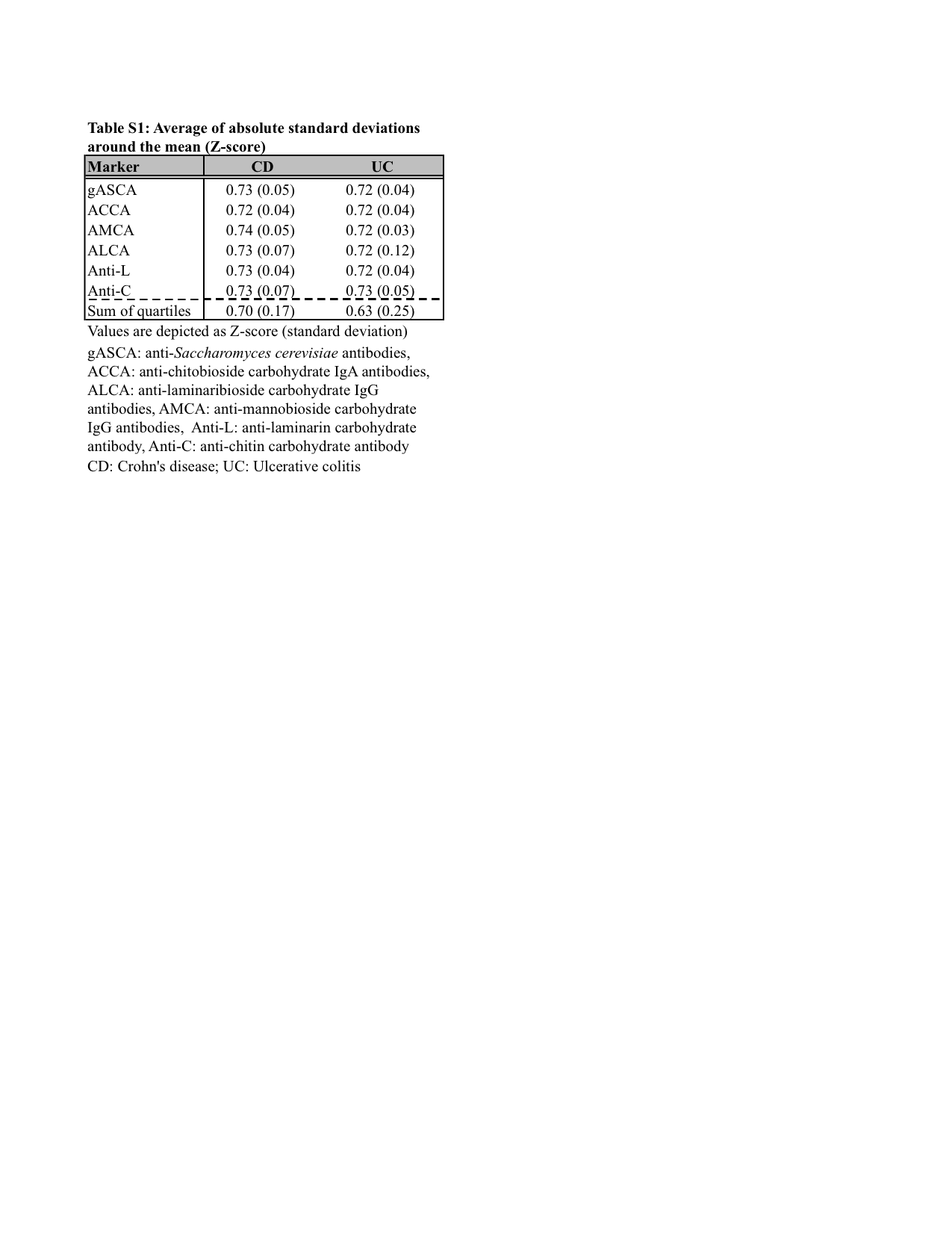

Supplement: Table S1 — Average of absolute standard deviations around the mean (Z-score). (TIFF) [file pone.0018172.s004.tiff]

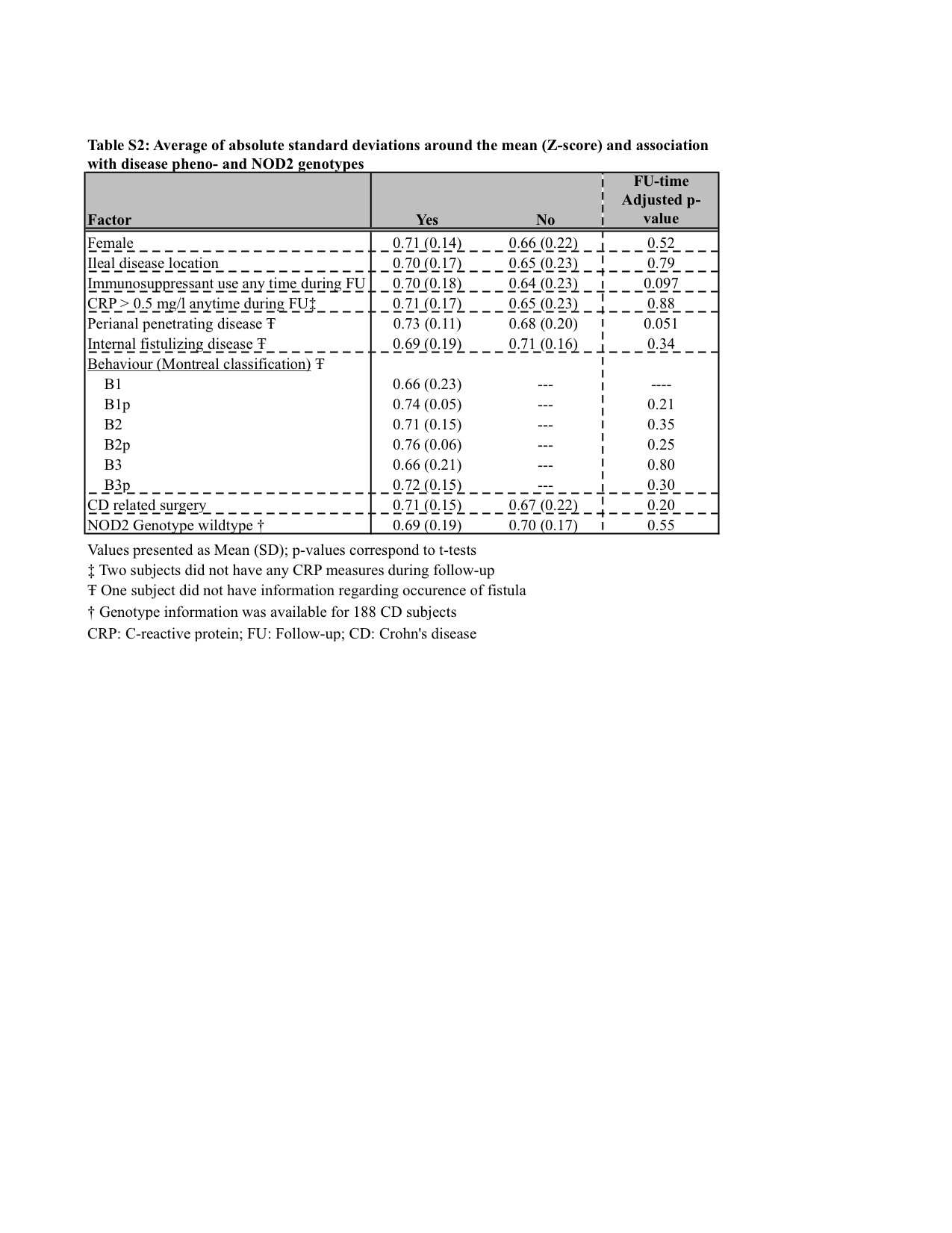

Supplement: Table S2 — Average of absolute standard deviations around the mean (Z-score) and association with disease pheno- and NOD2 genotypes. (TIFF) [file pone.0018172.s005.tiff]

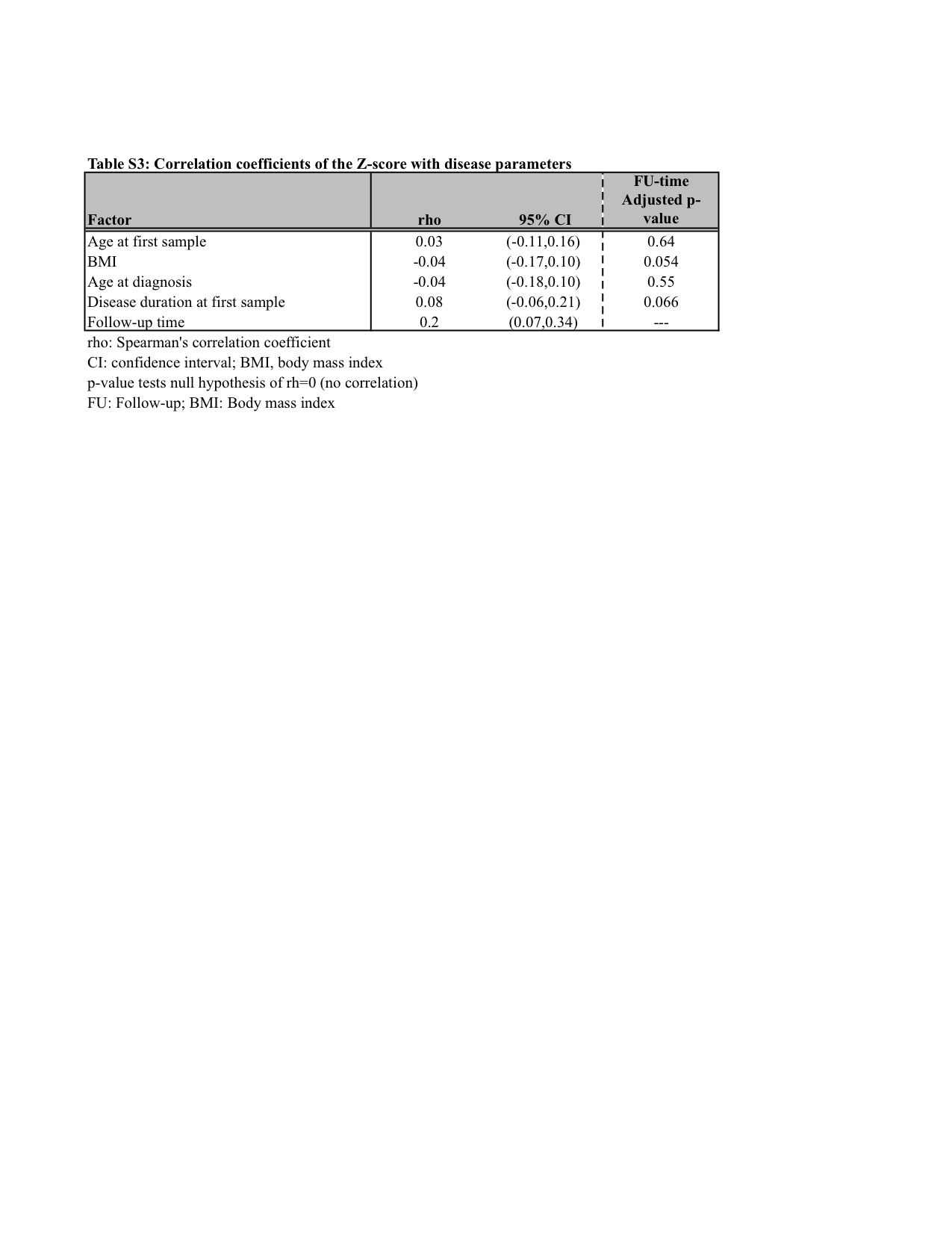

Supplement: Table S3 — Correlation coefficients of the Z-score with disease parameters. (TIFF) [file pone.0018172.s006.tiff]

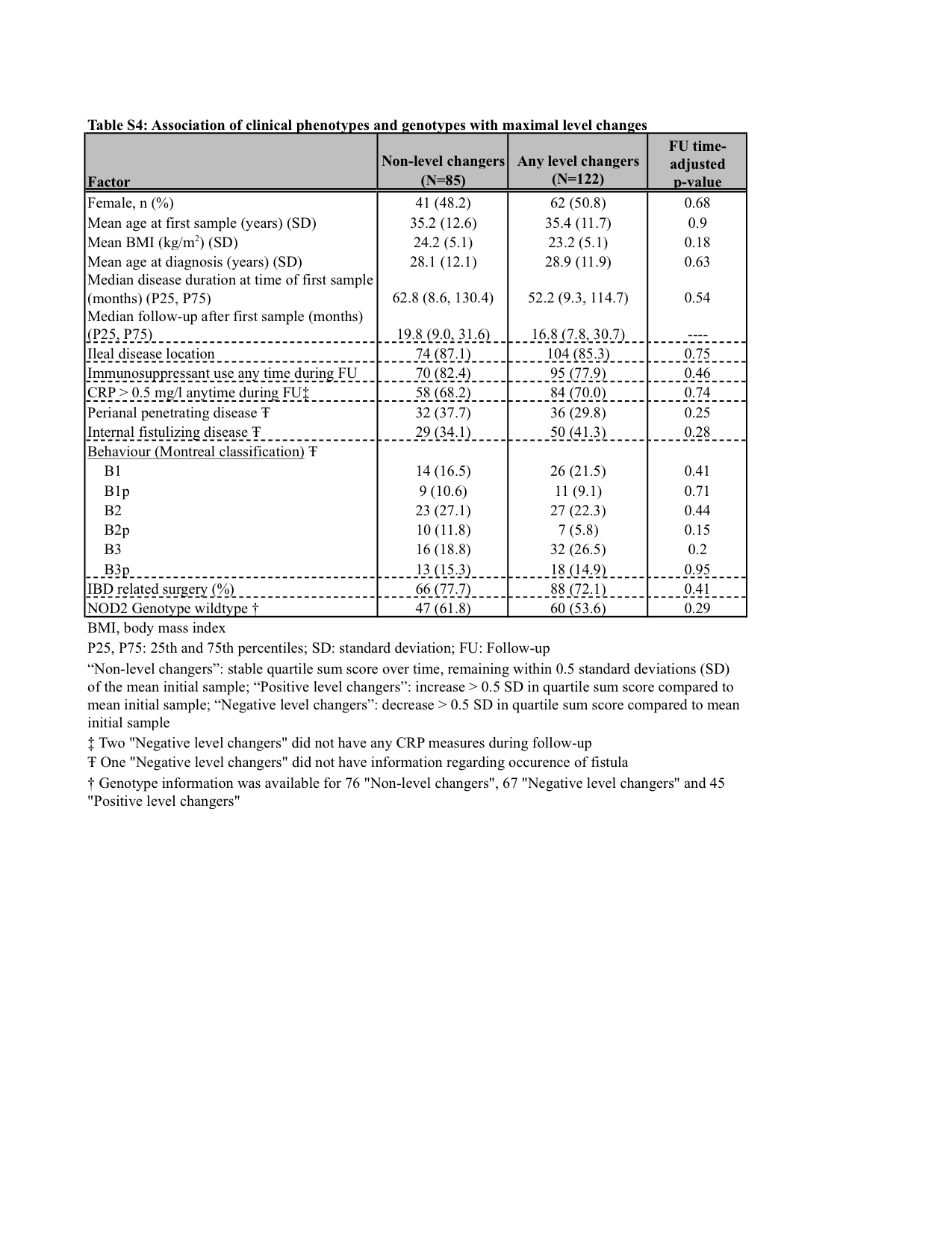

Supplement: Table S4 — Association of clinical phenotypes and genotypes with maximal level changes. (TIFF) [file pone.0018172.s007.tiff]

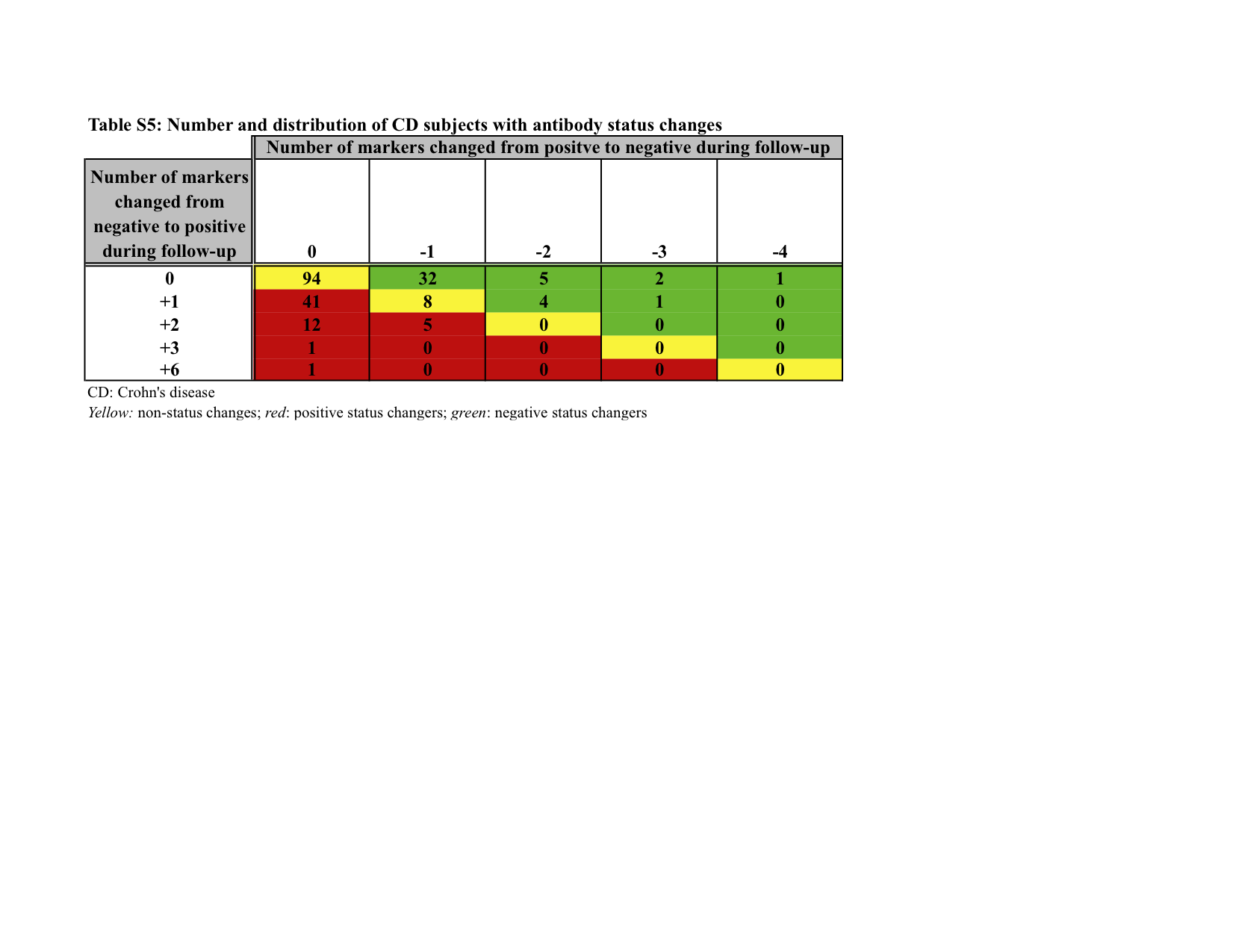

Supplement: Table S5 — Number and distribution of CD subjects with antibody status changes. (TIFF) [file pone.0018172.s008.tiff]

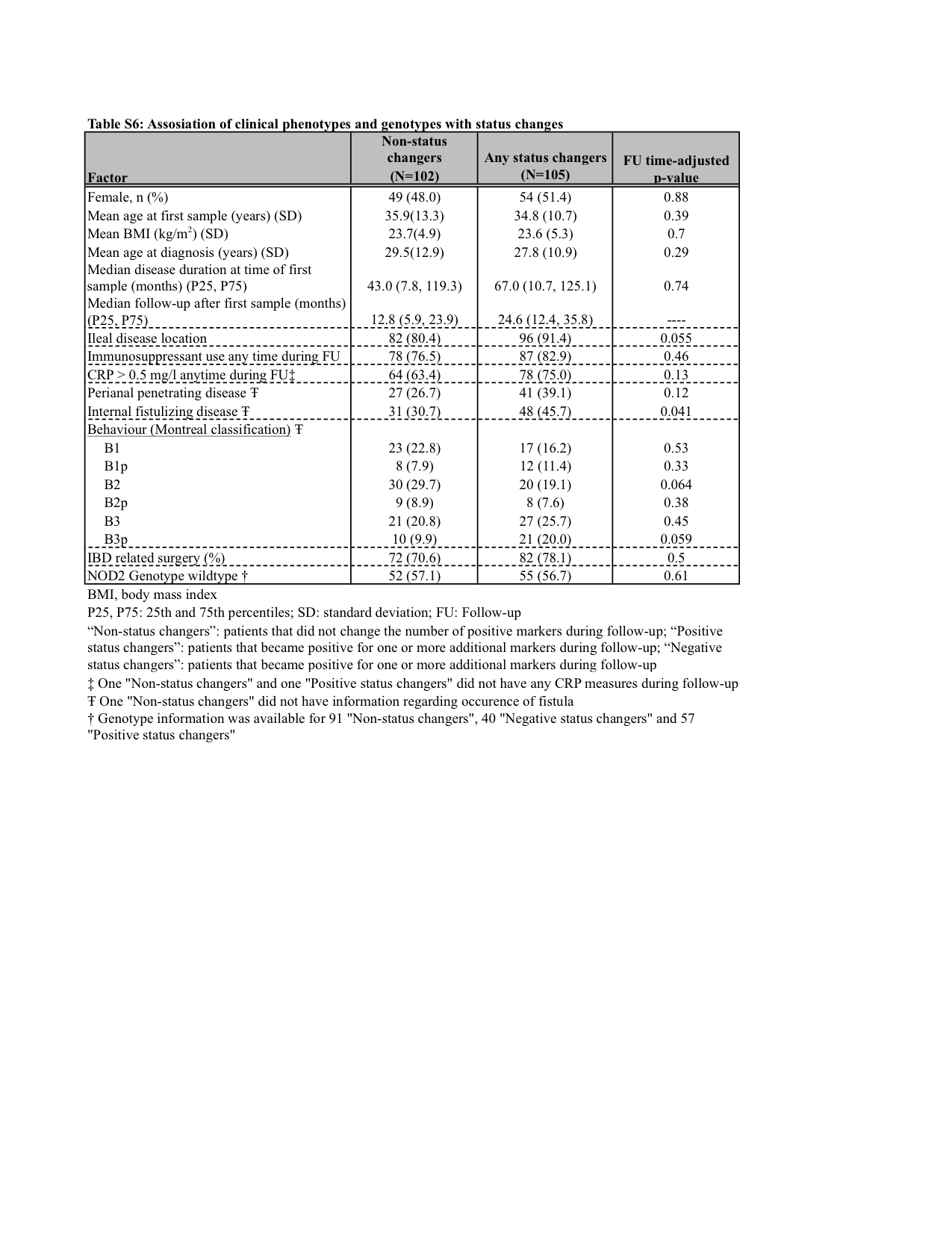

Supplement: Table S6 — Assosiation of clinical phenotypes and genotypes with status changes. (TIFF) [file pone.0018172.s009.tiff]

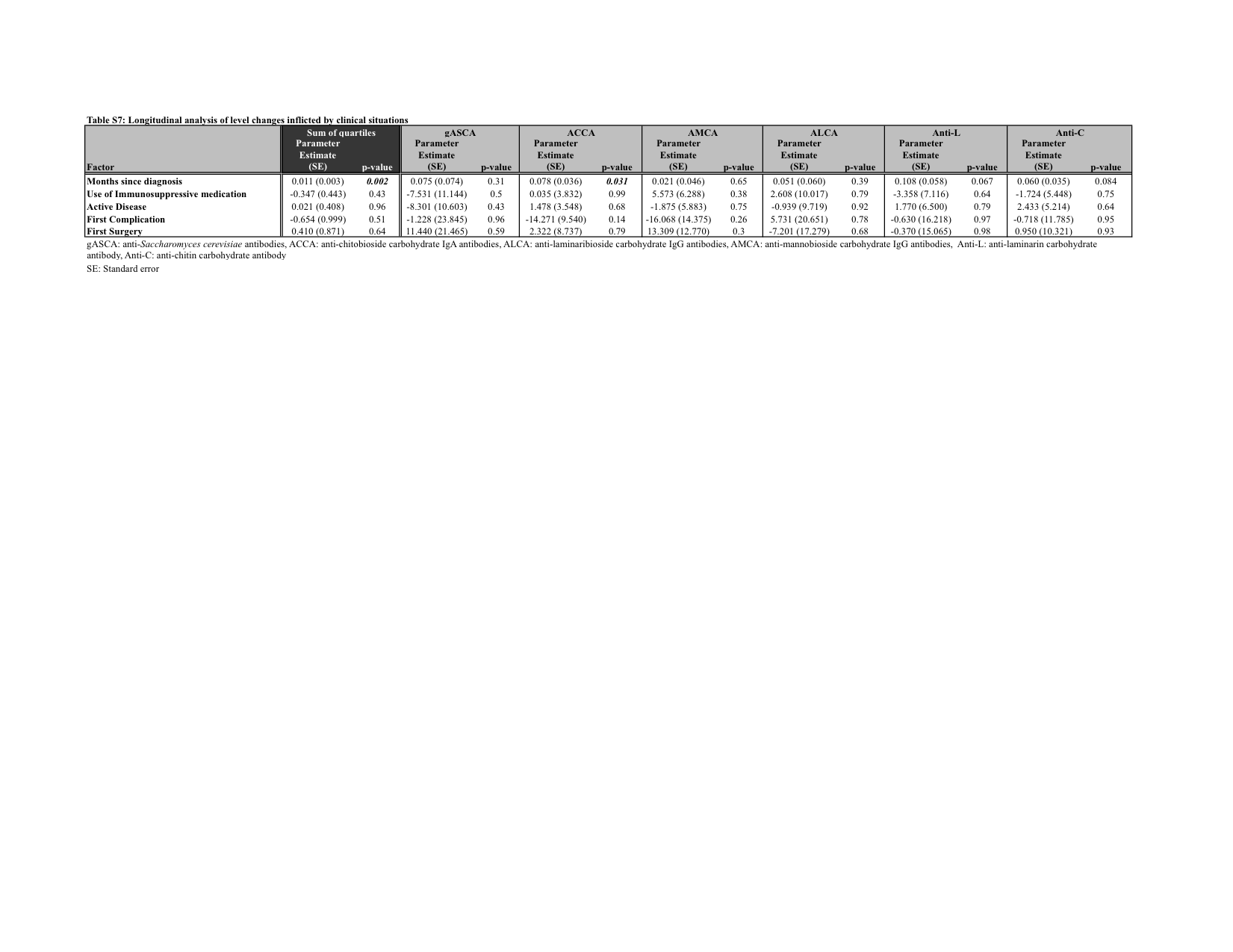

Supplement: Table S7 — Longitudinal analysis of level changes inflicted by clinical situations. (TIFF) [file pone.0018172.s010.tiff]

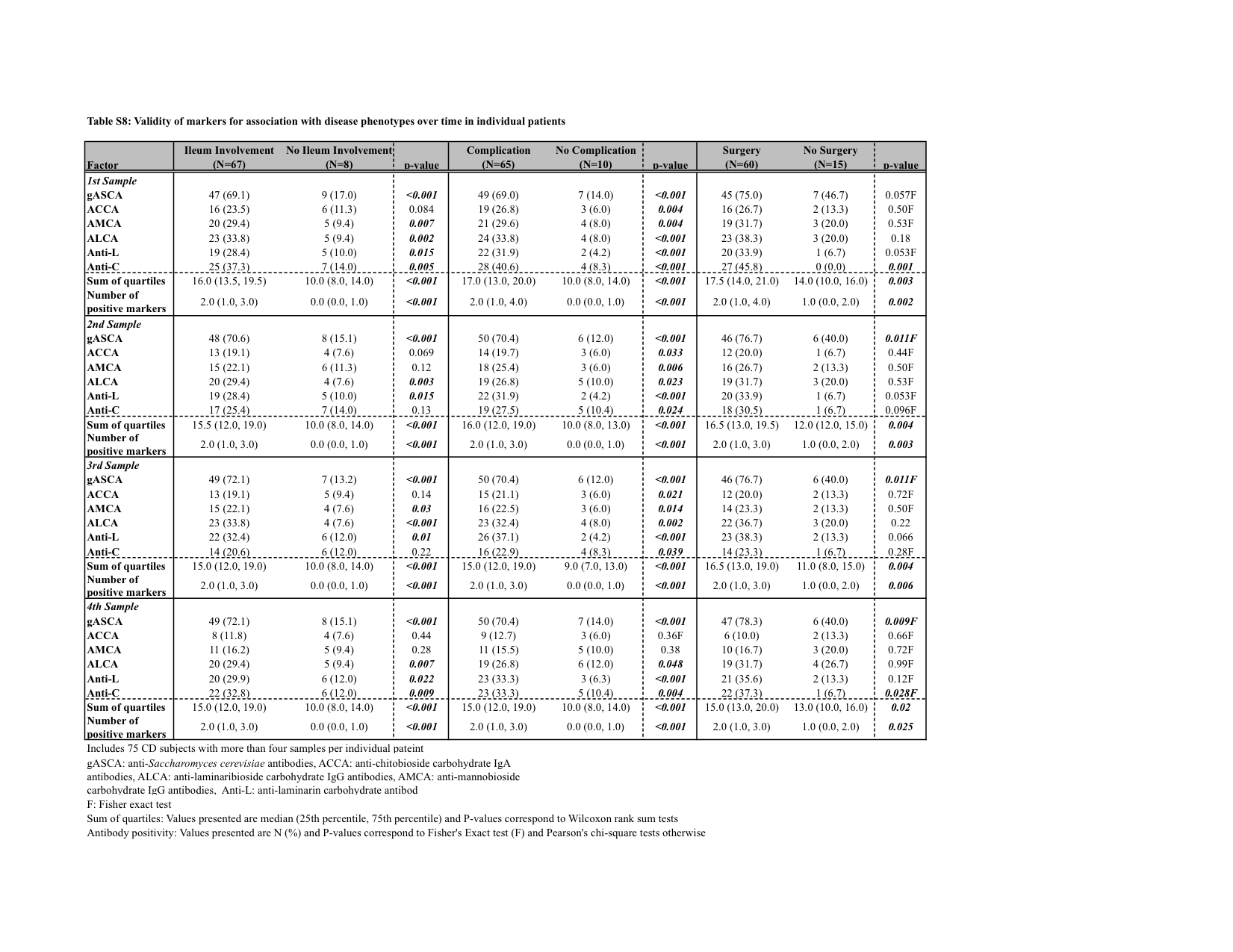

Supplement: Table S8 — Validity of markers for association with disease phenotypes over time in individual patients. (TIFF) [file pone.0018172.s011.tiff]
